# Computational intelligence-based investigation of heat transfer enhancement and entropy optimization in tri-hybrid nanofluid flow over a paraboloid needle

**DOI:** 10.1038/s41598-026-49041-w

**Published:** 2026-04-18

**Authors:** Javed Ahmad, Reem Abdullah Aljethi, Syed Asif Ali Shah, Jibran Hussain

**Affiliations:** 1 Department of Mathematics and Statistics, The University of Lahore, Lahore, Pakistan; 2 Department of Mathematics and Statistics, College of Science, Imam Mohammad Ibn Saud Islamic University (IMSIU), Riyadh, 13318 Saudi Arabia; 3 Zhejiang Qiaoshi Industry Co., Ltd., Ningbo, 315470 China

**Keywords:** Artificial neural network, Casson fluid, Thin needle, Entropy generation, Ternary nanofluid, Engineering, Mathematics and computing, Nanoscience and technology, Physics

## Abstract

Efficient heat transfer fluids are essential for contemporary thermal engineering, as traditional liquids insufficiently deliver the required cooling and heating efficacy. Hybrid and ternary nanofluids, owing to their superior thermo-physical properties, offer a promising alternative. However, their nonlinear rheological behavior and complex transport mechanisms pose substantial modeling difficulties. To address these difficulties, we proposed an artificial neural network (ANN)-based numerical approach to investigate the parameters influencing the heat transfer of a magnetized Casson-based $TiO_2 - MWCNT$ /ethylene glycol–water hybrid nanofluid and $TiO_2 - MWCNT - Al_2O_3$/ethylene glycol–water ternary nanofluid over a heated slender needle. We describe a new hybrid methodology that utilizes ANN and a modified Bvp4c approach to boost numerical modeling capabilities. We utilized similarity transformation to turn the governing partial differential equations into a set of ordinary differential equations. We use the MATLAB-based Bvp4c method to find numerical solutions to these equations. A parametric analysis is conducted to thoroughly examine the influence of essential dimensionless factors, including the nanoparticle shape factor *m*, the needle parameter *c*, the Casson parameter $\beta$, the magnetic parameter *M*, the radiation parameter *Rd*, and the thermophoresis parameter *Nt*. Quantitative analysis shows that the ternary nanofluid enhances the wall shear stress with a better skin friction coefficient than the hybrid nanofluid, though the local Nusselt number slightly decreases in the ternary nanofluid. Also, higher magnetic and Casson parameters cause higher flow resistance and reduced heat transfer, and higher thermal radiation improves temperature distribution. The ANN model shows a wide range of compatibility with numerical results and is a good alternative to an immediate thermal management system.

## Introduction

Artificial neural networks are computational models used in machine learning that are modeled after the human brain. These networks, which are utilized in processes such as recognition of images, machine learning, and time series prediction, are built up of connected nodes, or artificial neurons, that analyze input to discover patterns. Alqarni et al.^1^ employed an ANN technique to simulate the fluid movement and thermal transform of a Casson nanofluid between rotational disks. Afendi et al.^2^ forecasted subsurface temperatures using the ANN technique and temperature check survey data. Maatki et al.^3^ analyzed thermal transport in a tri-hybrid nanofluid thin film flow by using ANN techniques. Kanti et al.^4^ utilized experiments and machine learning techniques to examine how various mixture ratios impact thermal transport in hybrid nanofluids. Nasir et al.^5^ examined the impact of thermal transport of hybrid nanofluid flow through a porous cavity using ANN techniques. Wantao et al.^6^ utilized an improved artificial neural network to model the transient thermal transfer and flow characteristics of a complicated hybrid nanofluid, including temperature-dependent features.

Joshi et al.^7^ applied an ANN technique to forecast precisely and validate thermal transport characteristics in MHD micropolar fluid motion with bioconvection and radiation impacts. Ramasakher et al.^8^ used an ANN technique to authenticate and refine numerical results for MHD penta-hybrid nanofluid flow, enhancing forecast fidelity of skin friction and transport characteristics. Ramasekhar et al.^9^ employed a machine learning technique for forecasting thermal and mass transport in Maxwell-Sutterby fluid flow over a Riga plate via Levenberg–Marquardt backpropagation

A non-Newtonian fluid model characterized by yield stress is called the Casson fluid. This indicates that it behaves as a solid until the applied shear stress exceeds a specific threshold, at which juncture it flows like a fluid. The model was initially devised to represent the flow behavior of various complicated fluids, including blood (its most popular application), chocolate, printing inks, certain paints and colors, and specific suspensions and pastes. Tanveer et al.^10^ compared Levenberg-Marquardt and Bayesian Regularization neural networks for assessing heat transport in magnetized Casson fluid across various surfaces. Bejawada et al.^11^ examined a nonlinear inclined stretched porous surface with an MHD Casson fluid flow. Jalili et al.^12^ explored that a non-Darcy Casson fluid flow across a vertical stretched plate was affected by thermodiffusion, electrical fields, and nonlinear thermal radiation. Ajithkumar et al.^13^ investigated the MHD peristaltic flow of a radiative Casson fluid in an inclined porous tube using chemical processes, heat production, and Hall currents. Khan et al.^14^ analyzed the heat and mass transfer of a constant Casson fluid flow through a porous material across a stretched surface. Abbas et al.^15^ used the Atangana-Baleanu fractional derivative to evaluate Casson fluid flow with thermal/chemical effects, employing Laplace transforms and neural networks as solutions. Ramasekhar et al.^16^ investigated MHD Casson hybrid nanofluid (gold–silver in blood) flow over a stretching surface, highlighting its biomedical relevance in drug delivery.

A thin needle is a very thin instrument (similar to a syringe needle or a thin wire) intended to study how fluids travel and circulate heat around small-scale objects. Examples of tiny needles are a hypodermic needle flowing through blood (called Casson fluid), air velocity measured using a hot-wire anemometer, and optical fibers or synthetic threads coated accordingly. Bouzidi et al.^17^ employed response surface methodologies in analyzing optimal heat transfer in buoyancy-driven Williamson tri-hybrid nanofluid flow through a narrow needle. Samat et al.^18^ aimed to conduct a numerical and computational evaluation of hybrid carbon nanotubes traversing a vertical slender needle under suction. Afridi et al.^19^ investigated the thermal transmission and entropy production in an incompressible fluid along a slender needle surface. Hajlaoui et al.^20^ analyzed the velocity and thermal transport of a Maxwell nanofluid over a moving, variable-thickness thin needle within a porous media subjected to a magnetic field. Jena et al.^21^ tested the Casson fluid flow containing gyrotactic microorganisms across a thin needle in the presence of a magnetic field.

Entropy production is a thermodynamic measure that assesses the irreversibility of physical processes inside a system. It denotes the amount of valuable energy irreversibly lost owing to dissipative phenomena, such as viscous friction, thermal conduction over finite temperature gradients, mass diffusion, Joule heating, and chemical processes. Shah et al.^22^ analyzed enhancement of heat transfer of SiC and SiC-Ag hybrid nanofluids in the presence of methanol-based fluid over a stretching cylinder in when MHD, thermal radiation, and Darcy-Forchheimer effects on heat transfer are in place. Awan et al.^23^ studied that gyrotactic microorganisms increase mixing and greatly enhance convective heat transfer in a Riga plate Eyring powder nanofluid. Hassan et al.^24^ investigated entropy generation within a magnetohydrodynamic thermal system featuring a nano-encapsulated phase change material suspension. Iqbal et al.^25^, Necib et al.^26^ examined the entropy production analysis of ternary nanofluid through a thin needle. Afridi et al.^27^ examined computational and ANN-based entropy production examination of ternary nanofluid via a heated thin needle. Ramasekar^28^ discussed that the rising of the thermal radiation parameter enhances the temperature profile of the $Cu-Al_2O_3$ hybrid nanofluid using different geometries by using the Midrich method for heat transfer analysis.

A ternary nanofluid is the type of mixture obtained through the scattering of three types of nanoparticles in a fluid. The overall effects of this combination are to enhance the heat conduction, the rate of thermal transport, and the rheological properties as compared to single-particle or hybrid nanofluids. The heat and fluid flow applications of nanoparticles are better served by ternary nanofluids because of the enhanced diffusion of energy by the synergistic interplay of various shapes, sizes, and even materials of nanoparticles and the elimination of thermal resistance. Ternary nanofluids are used increasingly in advanced heat transfer systems and cooling technology and energy engineering. Adun et al.^29^ concluded that ternary nanofluids, which include three categories of nanoparticles, are superior in thermal transport compared to hybrid nanofluids. Sheikholeslami et al.^30^ examined a solar panel equipped with an innovative cooling duct incorporating ternary nanofluid, in conjunction with a thermoelectric generator, aimed at enhancing efficiency and thermal regulation. Manjunatha et al.^31^ presented a new tri-hybrid nanofluid theoretical model, exhibiting superior thermal transport compared to traditional hybrid nanofluids via mathematical formulation and numerical analysis. Smida et al.^32^ studied the computational thermal study, and Adun et al.^33^ studied the numerical exergy analysis of tri-hybrid nanofluid. Pemmaraju et al.^34^ integrated a machine learning methodology with computational fluid dynamics to describe ternary nanofluid electromagentohydrodynamic flow with heat generation.

The literature is deficient in a comprehensive study that amalgamates ANN-based prediction with entropy-optimized heat transfer in Casson tri-hybrid nanofluid flow across paraboloid needle geometries. This study bridges the gap by integrating non-Newtonian modeling, sophisticated nanofluid formulation, entropy analysis, and machine learning-driven prediction into a unified framework. The current work examines the heat transfer properties and stable boundary-layer flow of a $TiO_{2}-MWCNT$/ethylene glycol–water hybrid nanofluid across a heated thin needle with a paraboloid form. Casson fluid rheology and similarity transformations are used to develop the governing nonlinear differential equations. The Bvp4c solver in MATLAB yields a reliable numerical solution. Furthermore, an ANN model is created to forecast important thermal metrics such as skin friction, temperature profiles, velocity gradients, and Nusselt numbers. The accuracy, dependability, and generalizability of the ANN predictions are evaluated by comparing them with numerical outcomes.

This article is drafted to provide a brief and comprehensive analysis of the topic. Section “Applications" is about the use of the nanofluid model in engineering and thermal fields. Section “Novelty" presents the identifying characteristics of the present study in tandem with that of other studies and its contribution to the field. Section “Mathematical formulation" includes a mathematical explanation of the model. The numerical methods address the strategies and calculating tools used in the numerical techniques in Sect. “Numerical scheme". In Sect. “Irreversibility analysis", the analysis of irreversibility is given. Section “Results and discussion" gives an in-depth analysis of the calculated results and how the different physical parameters affect the velocity, temperature, concentration, and entropy generation profiles. This Sect. “Artificial neural network modeling" deals with the artificial neural network model, which describes the process of construction and training, as well as evaluation. Section “Comparison btween ANN and Bvp4c results" reviews the accuracy and reliability of predictions made by ANN by comparing them to numerical data. Section “Conclusion" draws the main conclusions of the research and its implications for the engineering and physical domain.

## Applications

**Fig. 1:**
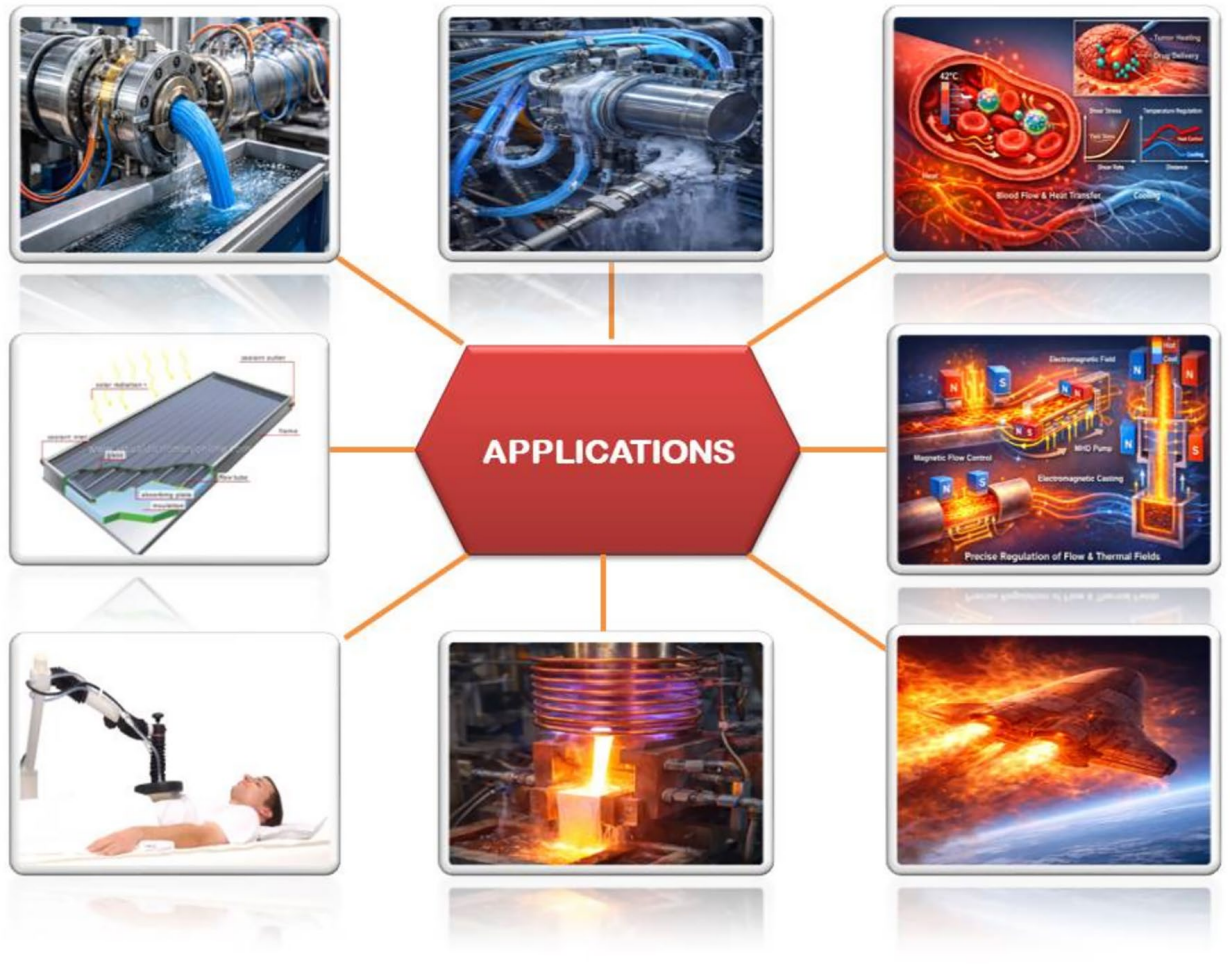
Applications.

It is shown that Casson-based ternary nanofluids exhibit non-Newtonian yield stress behavior, in addition to high thermal conductivity because of the combined effects of three dissimilar nanoparticles, thus being suitable in enhanced thermal transport. Biomedical systems use them to mimic blood-like flow during the drug delivery process in hyperthermia treatments and enhance the thermal performance of micro- and nano-scale electronic cooling devices, including solar collectors and heat exchangers, to increase their thermal efficiency. Furthermore, these nanofluids find application in magnetohydrodynamic flow regulation, industrial coating technologies, and aerospace thermal management, where accurate control of flow and superior heat transfer performance are essential (Fig. 1).

## Novelty

**Fig. 2:**
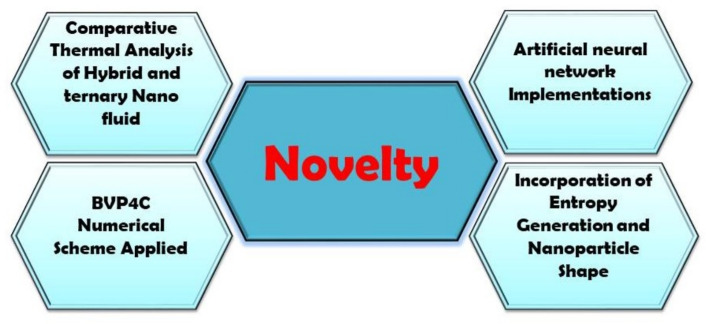
Novelty.

This paper advances the modeling of Casson-based ternary nanofluid flow over a heated slender needle, both in methodology and in physical insight. Whereas earlier studies focused on hybrid nanofluid flow and heat transfer in inclined porous cylinders, the present work contrasts Casson-based hybrid and ternary nanofluids by simultaneously accounting for thermal radiation, entropy generation, and nanoparticle shape factor. A hybrid ANN–Bvp4c approach is formulated to examine magnetized Casson-based $TiO_2\!-\!MWCNT$ integrated with a blend of ethylene glycol and water to create a hybrid and $TiO_2\!-\!MWCNT\!-\!Al_2O_3$ is in combination with a concoction of ethylene glycol and water to form a ternary nanofluid that passes over a heated fine needle. The model incorporates in particular nanoparticle shape factor, entropy production, magnetic field, and thermal radiation effects. The data indicate that ternary nanofluid provides better heat transfer than the hybrid nanofluid, that magnetic forces reduce the flow velocity, and that thermal radiation elevates the temperature profile. ANN-BVP4c hybrid method combines the efficiency of machine learning with numerical accuracy in predicting the results of the model at the level of parameter space in seconds without unnecessary repeated calculations. It supplements accuracy of standalone ANN models with physics-based training data, which results to better computational efficiency, generalization, and reliability, hence making it more efficient in parametric analysis and optimization (Fig. 2).

## Mathematical formulation

Present work analyzes the continuous, incompressible boundary layer flow of a tri-hybrid nanofluid composed of $Al_2O_3$, TiO_2_, and MWCNTs mixed with ethylene glycol and water as it traverses a heated paraboloid-shaped thin needle. MWCNTs have exceptionally high thermal conductivity and heat transfer is greatly advanced, unlike TiO_2_ which is highly resistant to corrosion and offers outstanding chemical stability to enable high long term reliable operation. Integration involves the addendum of $Al_2O_3$ to enhance dispersion and stability besides cost-effectiveness. The needle’s axis is oriented in the direction of the try-hybrid nanofluid flow, and its thickness is presumed to be comparable to or less than the boundary layer thickness. Pressure fluctuations along the needle surface are deemed insignificant. The needle is either at rest or in motion with a uniform velocity $u_w$ as the free-stream velocity $u_\infty$ of the tri-hybrid nanofluid. The needle’s temperature $T_w$ surpasses the ambient temperature $T_\infty$ of the surrounding fluid, i.e. $T_w > T_\infty$ (Fig. 3).

A cylindrical coordinate system $(x, r)$ is adopted, with the $x$-axis aligned along the axis of the paraboloid needle. The needle’s surface profile is defined by:

**Figure:**
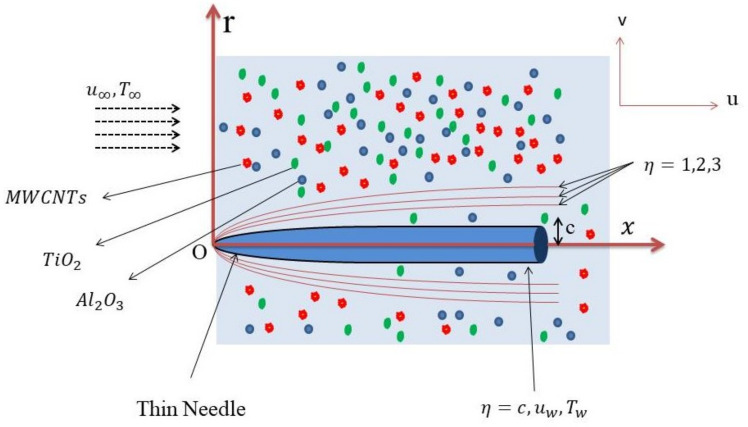
Boundary-layer flow past a paraboloid-shaped needle in ternary nanofluid.

1 $$\begin{aligned} R(x) = \left( \frac{\nu _f c x}{U} \right) ^{\frac{1}{2}}, \end{aligned}$$

where $U = u_w + u_\infty$ represents the composite velocity, the kinematic viscosity of water-ethylene glycol is, $\nu _f$ and $c$ denotes a characteristic size parameter of the needle.

The non-Newtonian behavior of the hybrid nanofluid is captured using the Casson fluid model. The constitutive equation for the Casson fluid under isotropic and incompressible flow conditions is expressed as

2 $$\begin{aligned} \tau _{ij} = {\left\{ \begin{array}{ll} 2 \left( \mu _B + \dfrac{p_y}{\sqrt{2\pi }} \right) e_{ij}, & \pi \ge \pi _c, \\ 2 \left( \mu _B + \dfrac{p_y}{\sqrt{2\pi _c}} \right) e_{ij}, & \pi < \pi _c. \end{array}\right. } \end{aligned}$$

Here, $\tau _{ij}$ denotes component of stress tensor, $e_{ij}$ is corresponding component of the deformation rate tensor, and $\pi = e_{ij} e_{ji}$ denotes the product of the deformation rate components. The parameters $\mu _B$ $p_y$ refer to dynamic viscosity and the yield stress of base fluid, respectively. A dimensionless Casson parameter $\beta = \mu _B \sqrt{2\pi _c}/p_y$ is introduced, which characterizes the reciprocal of the yield stress effect.

Using Prandtl’s estimations of the boundaries, we can write the equations that describe how the hybrid and ternary nanofluid flows and carries heat around the thin needle:

3 $$\begin{aligned}&\frac{\partial (ru)}{\partial x} + \frac{\partial (rv)}{\partial r} = 0, \end{aligned}$$

4 $$\begin{aligned}&u\frac{\partial u}{\partial x} + v\frac{\partial u}{\partial r} = \frac{\mu _{thnf}}{\rho _{thnf}} \left( 1 + \frac{1}{\beta } \right) \frac{1}{r} \frac{\partial }{\partial r} \left( r \frac{\partial u}{\partial r} \right) -\frac{\sigma _{thnf}B^{2}_{0}u}{\rho _{thnf}}, \end{aligned}$$

5 $$\begin{aligned}&u\frac{\partial T}{\partial x} + v\frac{\partial T}{\partial r} = \frac{1}{(\rho C_p)_{thnf}}\left( \frac{16\sigma ^{*}T^{3}_{\infty }}{3K^{*}} + k_{thnf} \right) \frac{1}{r} \frac{\partial }{\partial r} \left( r \frac{\partial T}{\partial r} \right) + \tau \left( D_{B}\frac{\partial T}{\partial r}\frac{\partial C}{\partial r}+\frac{D_{T}}{T_{\infty }}\left( \frac{\partial T}{\partial r}\right) ^{2}\right),\end{aligned}$$

6 $$\begin{aligned}&u\frac{\partial C}{\partial x} + v\frac{\partial C}{\partial r} = \frac{D_{B}}{r} \frac{\partial }{\partial r} \left( r \frac{\partial C}{\partial r} \right) + \frac{D_{T}}{T_{\infty }} \frac{1}{r}\frac{\partial }{\partial r} \left( r \frac{\partial T}{\partial r} \right). \end{aligned}$$

In the aforementioned equations, $\kappa _{thnf}, \mu _{thnf},$ and $\rho _{thnf}$ represent the coefficient of thermal conductivity, the dynamic viscosity, and the density of the tri-hybrid nanofluid.

The boundary conditions regulating the transport of boundary layer nanofluid at the surface of the slender needle and inside the surrounding nanofluid are specified as follows:

7 $$\begin{aligned} \begin{aligned} {\left\{ \begin{array}{ll} & \quad T = T_{w}, \quad u = u_{w},\quad D_{B}\frac{\partial T}{\partial r}+\frac{D_{T}}{T_{\infty }}\frac{\partial C}{\partial r}, \quad v = 0 \quad \text {at} \quad r = R(x), \\ & \quad T \rightarrow T_{\infty }, \quad u \rightarrow u_{\infty }, \quad C \rightarrow C_{\infty } \quad \text {as} \quad r \rightarrow \infty . \end{array}\right. } \end{aligned} \end{aligned}$$

We follow a sequential mixing approach: add base fluid *f*, add $\text {TiO}_2$ ($\gamma _1$) to get single-particle nanofluid ($\text {nf}$), add MWCNTs ($\gamma _2$) to get hybrid ($\text {hnf}$), and optionally add $\text {Al}_2\text {O}_3$ ($\gamma _3$) to obtain the ternary mixture ($\text {tern}$). Total volume-fraction constraint:

8 $$\begin{aligned} \gamma _1+\gamma _2+\gamma _3<1. \end{aligned}$$

After adding $\text {TiO}_2$ properties of nanofluid:

9 $$\begin{aligned} {\left\{ \begin{array}{ll} & \mu _{nf} = \frac{\mu _f}{(1 - \gamma _1)^{2.5}}, \\ & \rho _{nf} = (1 - \gamma _1)\rho _f + \gamma _1\rho _{s_1}, \\ & (\rho C_p)_{nf} = (1 - \gamma _1)(\rho C_p)_f + \gamma _1(\rho C_p)_{s_1}, \\ & \kappa _{nf} = \frac{\kappa _{s_1} + 2\kappa _f - 2\gamma _1\left( \kappa _f - \kappa _{s_1}\right) }{\kappa _{s_1} + 2\kappa _f + \gamma _1\left( \kappa _f - \kappa _{s_1}\right) }\kappa _f,\\ & \frac{\sigma _{nf}}{\sigma _{f}} = \frac{(1 + 2\phi _1)\sigma _{r1} + (1 - 2\phi _1)\sigma _{f}}{(1 - \phi _1)\sigma _{r1} + (1 + \phi _1)\sigma _{f}}. \end{array}\right. } \end{aligned}$$

Subsequently, the effective properties of the TiO$_2$-MWCNTs/EG-water hybrid nanofluid are computed:

10 $$\begin{aligned} {\left\{ \begin{array}{ll} & \mu _{hnf} = \frac{1}{(1 - \gamma _1)^{2.5}(1 - \gamma _2)^{2.5}}\mu _f, \\ & \rho _{hnf} = \left\{ (1 - \gamma _2)\left[ (1 - \gamma _1) + \gamma _1\frac{\rho _{s_1}}{\rho _f}\right] + \gamma _2\frac{\rho _{s_2}}{\rho _f}\right\} \rho _f, \\ & (\rho C_p)_{hnf} = \left\{ (1 - \gamma _2)\left[ (1 - \gamma _1) + \gamma _1\frac{(\rho C_p)_{s_1}}{(\rho C_p)_f}\right] + \gamma _2\frac{(\rho C_p)_{s_2}}{(\rho C_p)_f}\right\} (\rho C_p)_f, \\ & \kappa _{hnf} = \left( 1 - \gamma _2 + 2\gamma _2 \left( \frac{\kappa _{s_2}}{\kappa _{s_2}-\kappa _{nf}} \right) \ln \left( \frac{\kappa _{s_2}+\kappa _{nf}}{2\kappa _{nf}} \right) \right) \kappa _{nf},\\ & \frac{\sigma _{hnf}}{\sigma _{nf}} = \frac{(1 + 2\phi _2)\sigma _{r2} + (1 - 2\phi _2)\sigma _{nf}}{(1 - \phi _2)\sigma _{r2} + (1 + \phi _2)\sigma _{nf}}. \end{array}\right. } \end{aligned}$$

After adding $\text {Al}_2\text {O}_3$ ternary properties, $\text {tern}$ Treat the hybrid $\text {hnf}$ as the base when adding $\phi _3$ of $\text {Al}_2\text {O}_3$:

11 $$\begin{aligned} {\left\{ \begin{array}{ll} & \mu _{tern}=\frac{\mu _f}{(1-\gamma _1)^{2.5}(1-\gamma _2)^{2.5}(1-\gamma _3)^{2.5}}, \\ & \rho _{tern}=\Big \{(1-\gamma _3)\Big [(1-\gamma _2)\Big [(1-\gamma _1)+\gamma _1\frac{\rho _{s_1}}{\rho _f}\Big ]+\gamma _2\frac{\rho _{s_2}}{\rho _f}\Big ]+\gamma _3\frac{\rho _{s_3}}{\rho _f}\Big \}\rho _f, \\ & (\rho C_p)_{tern}=\Big \{(1-\gamma _3)\Big [(1-\gamma _2)\Big [(1-\gamma _1)+\gamma _1\frac{(\rho C_p)_{s_1}}{(\rho C_p)_f}\Big ]+\gamma _2\frac{(\rho C_p)_{s_2}}{(\rho C_p)_f}\Big ]+\gamma _3\frac{(\rho C_p)_{s_3}}{(\rho C_p)_f}\Big \}(\rho C_p)_f, \\ & \kappa _{tern}=\Big (1-\gamma _3+2\gamma _3\frac{\kappa _{s_3}}{\kappa _{s_3}-\kappa _{hnf}}\ln \!\Big (\frac{\kappa _{s_3}+\kappa _{hnf}}{2\kappa _{hnf}}\Big )\Big )\,\kappa _{hnf},\\ & \frac{\sigma _{tnf}}{\sigma _{hnf}} = \frac{(1 + 2\phi _3)\sigma _{r3} + (1 - 2\phi _3)\sigma _{hnf}}{(1 - \phi _3)\sigma _{r3} + (1 + \phi _3)\sigma _{hnf}}. \end{array}\right. } \end{aligned}$$

The physical properties of nanoparticles ($\text {TiO}_2$, MWCNTs, $\text {Al}_2\text {O}_3$), and base fluid (water–ethylene glycol) are shown in Table 1.

**Table 1:** Physical properties of nanoparticals ($\text {TiO}_2$, MWCNTs, $\text {Al}_2 \text {O}_3$), and base fluid (water–Ethylene Glycol mixture).

Physical properties	$\rho$ (kg/m$^{3}$)	$C_p$ (J/kg K)	$\kappa$ (W/m K)	$\sigma$ (S/m)
TiO$_2$	4250	686.2	8.9538	2.6 $\times 10^{6}$
MWCNTs	1600	796	3000	5 $\times 10^{6}$
Al$_2$O$_3$	3970	765	30	$\approx 0$
Water–Ethylene Glycol	1083	2824	0.316	5.03 $\times 10^{-4}$

The non-dimensional similarity variable ($\eta$) and stream function ($\psi$) are defined as We define the following similarity transformations.

12 $$\begin{aligned} {\left\{ \begin{array}{ll} \eta = \frac{U r^2}{\nu _f x}, \qquad \qquad \psi = \nu _f x f(\eta ), \\ u = \frac{1}{r} \frac{\partial \psi }{\partial r} = \frac{U}{\nu _f x} \frac{\partial \psi }{\partial \eta } = U f'(\eta ), \qquad v = -\frac{1}{r} \frac{\partial \psi }{\partial x}.\\ \theta (\eta ) = \frac{T - T_\infty }{T_w - T_\infty } \qquad {\phi (\eta ) = \frac{C-C_\infty }{C_w-C_\infty }} \end{array}\right. } \end{aligned}$$

Applying these transformations yields the following non-dimensional momentum and energy equations:

13 $$\begin{aligned}&\mathcal {A} f f'' + \frac{2}{\mathcal {V}}\Big (1+\frac{1}{\beta }\Big )\big (\eta f''' + f''\big )-M B_{5}f'=0, \end{aligned}$$

14 $$\begin{aligned}&\frac{2}{Pr}\left( B_{4}+\frac{4}{3}Rd\right) \big (\theta ' + \eta \theta ''\big ) + \mathcal {B} \left( f \theta '+2\eta \left( Nb\theta '\phi '+Nt\theta '^{2}\right) \right) =0,\end{aligned}$$

15 $$\begin{aligned}&2 \big (\phi ' + \eta \phi ''\big ) + 2\frac{Nt}{Nb}\big (\theta ' + \eta \theta ''\big ) +Sc f \phi ' =0. \end{aligned}$$

where

16 $$\begin{aligned} {\left\{ \begin{array}{ll} \mathcal {A}& =(1-\gamma _3)\Big [(1-\gamma _2)\big ((1-\gamma _1)+\gamma _1\frac{\rho _{s_1}}{\rho _f}\big )+\gamma _2\frac{\rho _{s_2}}{\rho _f}\Big ]+\gamma _3\frac{\rho _{s_3}}{\rho _f},\\ \mathcal {B}& =(1-\gamma _3)\Big [(1-\gamma _2)\big ((1-\gamma _1)+\gamma _1\frac{(\rho C_p)_{s_1}}{(\rho C_p)_f}\big )+\gamma _2\frac{(\rho C_p)_{s_2}}{(\rho C_p)_f}\Big ]+\gamma _3\frac{(\rho C_p)_{s_3}}{(\rho C_p)_f},\\ \mathcal {V}& =(1-\gamma _1)^{2.5}(1-\gamma _2)^{2.5}(1-\gamma _3)^{2.5},\\ B_{4}& =\frac{\kappa _{thnf}}{\kappa _f}, \qquad B_{5}= \frac{\sigma _{thnf}}{\sigma _{nf}} \end{array}\right. } \end{aligned}$$

The dimensionless parameters are defined as

17 $$\begin{aligned} M = \frac{\sigma _{thnf}B_0^2}{a\rho _{thnf}} \qquad Rd = \frac{4\sigma T^3_\infty }{k_{thnf}k^*} \qquad Pr = \frac{\mu _{thnf}c}{k_{thnf}} \qquad Sc = \frac{\nu _f}{D_B} \qquad Nb = \frac{\tau D_B (C_w - C_\infty )}{\nu _f} \qquad Nt&= \frac{\tau D_T (T_w - T_\infty )}{\nu _f T_\infty } \end{aligned}$$

subject to the boundary conditions:

18 $$\begin{aligned} \begin{aligned} {\left\{ \begin{array}{ll} & f'(c) = \frac{\lambda }{2}, \quad f(c) = \frac{\lambda c}{2}, \quad \theta (c) = 1, \quad Nt \phi '(c)+Nb \theta '(c), \\ & f'(\eta ) \rightarrow \frac{1-\lambda }{2}, \quad \theta (\eta ) \rightarrow 0,\quad \phi (\eta ) \rightarrow 0 \quad \text {as} \quad \eta \rightarrow \infty . \end{array}\right. } \end{aligned} \end{aligned}$$

Local Nusselt number and skin friction

$$\begin{aligned}&q_w=-\kappa \Big (\frac{\partial T}{\partial r}\Big )_{r=R(x)}, \qquad&\tau _w=2\mu \Big (1+\frac{1}{\beta }\Big )\Big (\frac{\partial u}{\partial r}\Big )_{r=R(x)}. \end{aligned}$$

Non-dimensional forms used for reporting are

$$\begin{aligned}&Nu_x = \frac{x q_w}{k_f (T_w-T_\infty )},\qquad \qquad Re_x=\frac{U x}{\nu _f},\\&Nu_x Re_x^{-1/2} = -2B_{4} c^{1/2} \theta '(c), \qquad C_f Re_x^{1/2} = 8 c^{1/2}\Big (1+\frac{1}{\beta }\Big )\frac{1}{\mathcal {V}} f''(c). \end{aligned}$$

As before, choose $\mathcal {V}$ according to the hybrid or ternary case.

## Numerical scheme

To solve the transformed boundary value problem, we employ MATLAB’s Bvp4c solver. The governing similarity equations are recast into 1*st*-order ODEs. Let

$$z_1 = f, \quad z_2 = f', \quad z_3 = f'', \quad z_4 = \theta , \quad z_5 = \theta ', \quad z_6 = \phi , \quad z_7 = \phi ', \quad zz_1 = f''', \quad zz_2 = \theta '', \quad zz_3 = \phi ''$$

The system becomes

19 $$\begin{aligned} {\left\{ \begin{array}{ll} zz_1 & = \frac{M B_5 z_2 - \mathcal {A} z_1 z_3 - \frac{2}{\mathcal {V}}\left( 1+\frac{1}{\beta }\right) z_3}{\frac{2}{\mathcal {V}}\left( 1+\frac{1}{\beta }\right) \eta }, \\ zz_2 & = \frac{-z_5\big [\frac{2}{\Pr }\left( B_4+\frac{4}{3}Rd\right) + \mathcal {B} z_1 + 2\mathcal {B}\eta (Nb\, z_7 + Nt\, z_5)\big ]}{\frac{2}{\Pr }\left( B_4+\frac{4}{3}Rd\right) \eta }, \\ zz_3 & = \frac{-(2 + Sc\, z_1) z_7 - 2\frac{Nt}{Nb} z_5 - 2\frac{Nt}{Nb} \eta zz_2}{2\eta }, \end{array}\right. } \end{aligned}$$

The boundary conditions are expressed as

20 $$\begin{aligned} {\left\{ \begin{array}{ll} \text {At} \eta = c: & \quad z_1(c) = \frac{\lambda c}{2}, \quad z_2(c) = \frac{\lambda }{2}, \quad z_4(c) = 1, \quad Nt\, z_7(c) + Nb\, z_5(c) = 0. \\ \text {As} \eta \rightarrow \infty : & \quad z_2(\infty ) \rightarrow \frac{1-\lambda }{2}, \quad z_4(\infty ) \rightarrow 0, \quad z_6(\infty ) \rightarrow 0. \end{array}\right. } \end{aligned}$$

The above system is solved using Bvp4c by constructing an initial mesh over the interval $\eta \in [c, \eta _{\max }]$, where $\eta _{\max }$ is chosen sufficiently large to approximate the far-field boundary conditions. A smooth initial guess $z_i(\eta )$ is provided so that the boundary conditions are satisfied approximately at both the needle surface and the far field. To ensure numerical stability and accuracy, the solver tolerances are specified using bvpset with RelTol$=10^{-6}$ and AbsTol$=10^{-8}$.

The engineering quantities are obtained from the numerical solution:

21 $$\begin{aligned} Nu_x Re_x^{-1/2}&= -2 B_{4} c^{1/2} z_5(c), \end{aligned}$$

22 $$\begin{aligned} C_f Re_x^{1/2}&= 8 c^{1/2}\left( 1+\frac{1}{\beta }\right) \frac{1}{\mathcal {V}} z_3(c). \end{aligned}$$

**Table 2:** Skin friction coefficient and the Nusselt number of hybrid and tri-hybrid nanofluids for various variable inputs.

*M*	$\beta$	*c*	*Rd*	$Re^{0.5}_{x}C_{f}$	$Re^{0.5}_{x}C_{f}$	$Re^{-0.5}_{x}Nu_{x}$	$Re^{-0.5}_{x}Nu_{x}$
				Hybrid case	Ternary case	Hybrid case	Ternary case
1.1	0.1	0.1	0.5	−0.5809	−1.6201	5.5958	5.1943
1.2				−0.5907	−1.6430	5.5845	5.1837
1.3				−0.6001	−1.6653	5.5736	5.1735
1.0	0.2			−0.7392	−1.7778	5.4076	5.1239
	0.3			−0.8527	−1.9072	5.2887	5.0715
	0.4			−0.9381	−2.0062	5.2072	5.0342
	0.1	0.2		−0.5982	−1.7806	3.6302	3.2741
		0.3		−0.6257	−1.9661	2.8661	2.5453
		0.4		−0.6535	−2.1531	2.4478	2.1547
		0.1	0.4	−0.5709	−1.5965	5.7407	5.3018
			0.6	−0.5709	−1.5965	5.4862	5.1185
			0.8	−0.5709	−1.5965	5.2736	4.9678

**Table 3:** Comparison of $f^{''}(c)$ for different values of *c*, $\beta \rightarrow \infty$ and values of all other parameters equal to zero.

*c*	Dharmaiah et al.^35^	Ishak et al.^36^	Current study
0.1	1.28881	1.2888	1.2886578
0.01	8.49244	8.4924	8.4932562
0.001	62.16372	62.1637	62.1641569

## Irreversibility analysis

The production of entropy is inherently connected to the loss of energy; thus, the reduction of entropy generation is what must be done to improve the thermodynamic performance of thermal systems. The analysis of entropy generation gives essential insights into the sources of irreversibility and helps to identify the main mechanisms of the appearance of the energy losses. Irreversibility in magnetohydrodynamic try-hybrid nanofluid motion is chiefly caused by heat transfer across constrained temperature gradients, viscous dissipation due to fluid friction, and Joule heating influenced by the applied magnetic field. The rate of local volumetric entropy generation in the presence of a magnetic field is stated as

23 $$\begin{aligned} E'''_{\text {gen}} = \frac{k_{thnf}}{T_\infty ^{2}}(\nabla T)^2 + \frac{1}{T_\infty }\left[ (\textbf{J}-QV)\times (\textbf{E}+\textbf{V}\times \textbf{B})\right] + \frac{\mu _{thnf}}{T_\infty }\Phi , \end{aligned}$$

where $k_{thnf}$, $\mu _{thnf}$, and $T_\infty$ denote the thermal conductivity, dynamic viscosity of the try-hybrid nanofluid, and ambient temperature, respectively. Here $\Phi$, $\nabla$, and $\textbf{J}$ are viscous dissipation, the Del operator, and current density, respectively.

The contribution of the electric field is disregarded because of an assumption of a low magnetic Reynolds number that suits a flow in the boundary layer of nanofluids and ensures that the main entropy-generating mechanisms are accurately determined. Ignoring the effects of the electrical field and charge velocity in relation to the magnet term $\textbf{V}\times \textbf{B}$, and utilizing surface layer approximations, Eq. (23) simplifies to

24 $$\begin{aligned} E'''_{\text {gen}} = \frac{\mu _{thnf}}{T_\infty }\left( \frac{\partial u}{\partial y}\right) ^2 + \frac{k_{thnf}}{T_\infty ^{2}}\left( \frac{\partial T}{\partial y}\right) ^2 + \frac{1}{T_\infty }\left( B_0^{2}\sigma _{thnf}u^{2}\right) , \end{aligned}$$

which indicates that entropy production occurs due to viscous dissipation, thermal transfer irreversibility, and magnetic dissipation.

The non-dimensional entropy generation rate is expressed as the ratio of the local entropy generation rate to the characteristic entropy production rate $E'''_0$.

25 $$\begin{aligned} N_s = \frac{E'''_{\text {gen}}}{E'''_0} = \theta '^2 + \text {Pr}\,\text {Ec}\, (f''^{\,2} + M\,f'^{\,2}), \end{aligned}$$

Where $E'''_0 = \dfrac{k_f (T_w-T_\infty )^2}{T_\infty ^{2}\nu _f}$ denotes the characteristic entropy generation rate. The Bejan numbers are defined as

$$\implies Be = \frac{\text {Heat transfer irreversibility}}{\text {Total irreversibility}} = \frac{\theta '^2}{N_s}$$

## Results and discussion

This section provides an extensive comparative analysis of hybrid and ternary nanofluids via ANN strategy, employing the Bvp4c method in MATLAB followed by the use of ANN through Python programming. Numerical results are validated with published studies for limiting cases. The impacts of significant parameters on velocity, temperature, concentration, Bejan numbers, and entropy generation are examined. Default values of utilized parameters are: $Nb=0.3, Nt=0.1, Le=0.2, Rd=0.5, c=0.1, \lambda =1, \phi _1=0.02, \phi _2=0.02, \phi _3=0.02, m=3, M=1,\beta =0.1, Ec=0.7$

### Numerical validation

The quantitative analysis of the impact of magnetic parameter, *M*, Casson parameter, $\beta$, needle parameter, *c* and radiation parameter, *Rd* on the skin friction coefficient $Re_x^{0.5}Cf$ and Nusselt number $Re_x^{-0.5}Nu_x$ of both hybrid and ternary nanofluid is indicated in Table 2. The analysis of the Nusselt number and skin fraction in Table 2 shows that the ternary nanofluid enhances the wall shear stress with a better skin friction coefficient than the hybrid nanofluid, though the local Nusselt number slightly decreases in the ternary nanofluid. This is a measure of a trade-off in enhanced momentum transport and thermal efficiency. Also, higher magnetic and Casson parameters cause higher flow resistance and reduced heat transfer, and higher thermal radiation improves temperature distribution. Analytical solutions to the nonlinear equations are unavailable. Thus, to determine reliability, extensive numerical validation is required. Table 3 shows a comparison of the $f''(c)$ at different values of the needle parameter in the limiting case of $\beta \rightarrow \infty$, where all other parameters are held at zero. The findings of the current investigation correspond to those of Dharmaiah et al.^35^ and Ishak et al.^36^, which proves the validity and accuracy of the existing numerical approach.

### Velocity profile

**Fig. 4:**
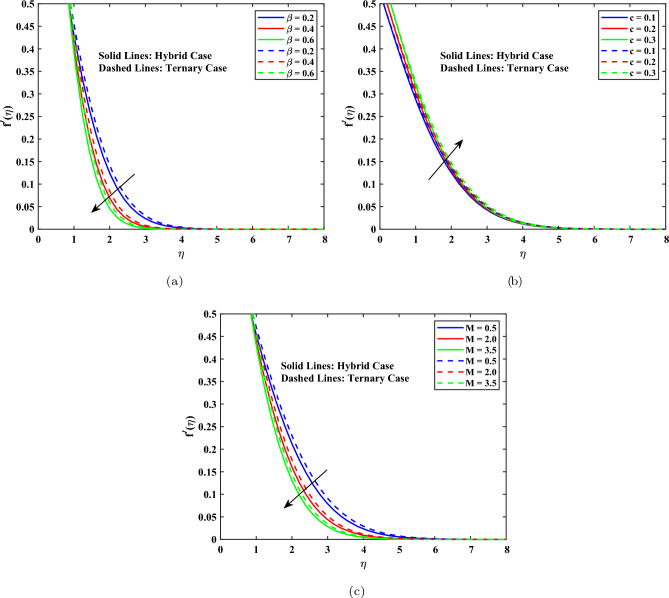
Velocity profile influenced by different parameters.

Figure 4 shows the effects of the Casson parameter $\beta$, the needle parameter *c*, and the magnetic parameter *M* on the non-dimensional velocity profile $f'(\eta )$ for both hybrid and ternary nanofluids. The hybrid nanofluid is represented by solid lines, and the ternary nanofluid is represented by dashed lines, which can be interpreted as the fact that a higher value of the Casson fluid parameter, denoted by $\beta$, corresponds to a large decrease in the velocity difference between the two nanofluid models, as can be seen in Fig. 4(a). Increasing the value of the $\beta$ enhanced yield stress of the Casson fluid results in the increment of the effective viscosity and the resistance to flow, which subsequently causes the reduction of the momentum boundary layer. Moreover, the synergistic effect of multiple nanoparticles implies that the ternary nanofluid will always have higher velocities than the hybrid nanofluid, which means that it has a better momentum transfer. Figure 4(b) indicates that the velocity profile decreases with an increase in the needle parameter *c* as the surface friction and viscous drag are higher with the increase in the size of the needles. This makes the thickness of the boundary layer smaller. The ternary nanofluid had a better velocity than the hybrid nanofluid, and this was an indication of the better rheological performance of the ternary nanofluid. As shown in Fig. 4(c), an increment in magnetic parameter, *M*, limits the flow to create an opposite Lorentz force that results in a decreased momentum barrier layer. The ternary nanofluid will always be better than the hybrid nanofluid in terms of velocity in the same circumstances. It means that ternary nanofluid exhibits decreased magnetic damping, which is likely to be explained by the fact that the nanoparticles interact with each other more effectively. The casson, needle, and magnetic parameters are easily increased to diminish the velocity field. The ternary nanofluid, in any case, is better than the hybrid nanofluid in terms of momentum transfer. Both the boundary conditions and the numerical solution are supported by the constant reduction in velocity to zero.

### Temperature profile

**Fig. 5:**
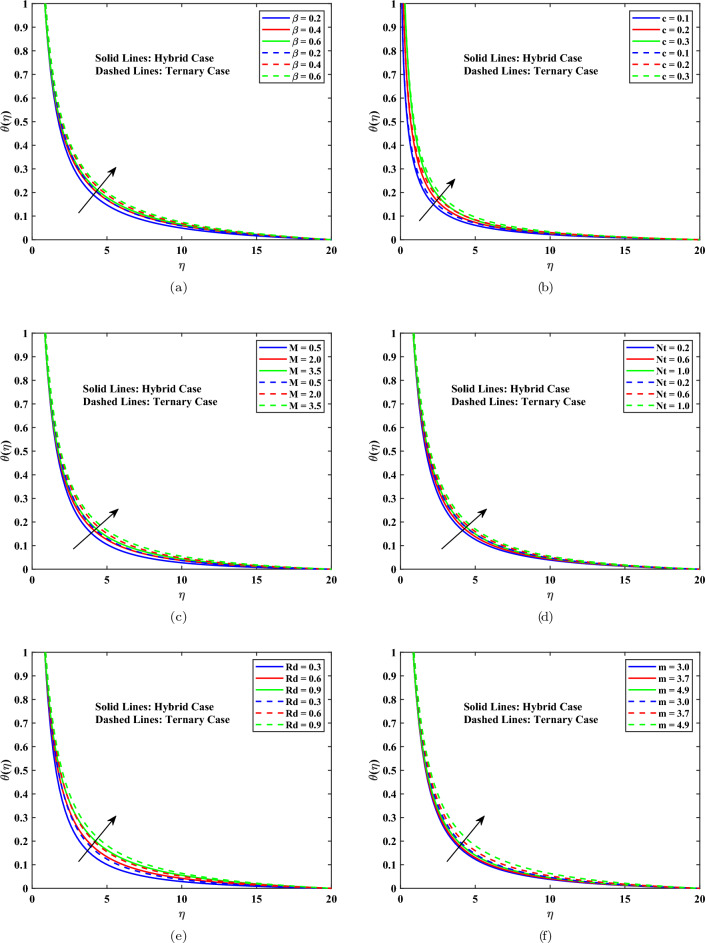
Temperature profile influenced by different parameters.

The impact of different parameters on the $\theta (\eta )$ of hybrid and ternary nanofluids is discussed here. Figure 5(a), illustrates the upper-level temperature distribution is enhanced by increasing the Casson fluid parameter, $\beta$. This action is caused by increased yield stress and efficient viscosity, which reduce convective heat transport and allow the buildup of thermal energy in the boundary layer. Figure5(b) shows that the higher the needle parameter, *c*, higher the surface friction and the lower the heat dissipation, and therefore the higher the temperature. As it is shown in Fig. 5(c) a rise in magnetic strength, *M*, leads to a significant increase in temperature. The Lorentz force curbs the movement of fluids and converts kinetic energy to thermal energy through Joule heating. Figure 5(d) shows that an increase in the thermophoresis parameter *Nt* produces significant enhancement of the temperature profile. This effect arises from nanoparticles’ thermophoretic mobility from hotter to cooler places, which increases energy transfer and thickens the thermal boundary layer. The effect of the radiation parameter. *Rd* is illustrated in Fig. 5(e). It has been observed that raising *Rd* produces a considerable rise in temperature. Physically, increased thermal radiation promotes radiative heat flow, rising fluid temperature, and rising thermal boundary layer thickness. Figure 5(f) shows that increasing the nanoparticle shape factor m enhances the temperature profile in hybrid and ternary nanofluids. Elevated m values indicate that non-spherical particles have a larger surface area, which improves effective thermal conductivity and increases heat transmission. Thermal energy accumulates in the boundary layer, increasing its thickness. The ternary nanofluid continuously exhibits slightly increased temperatures due to the nanoparticles’ synergistic effects. In all cases, the ternary nanofluid exhibited somewhat higher temperatures than the hybrid nanofluid, showing increased thermal conductivity due to many nanoparticle interactions.

### Concentration profile

**Fig. 6:**
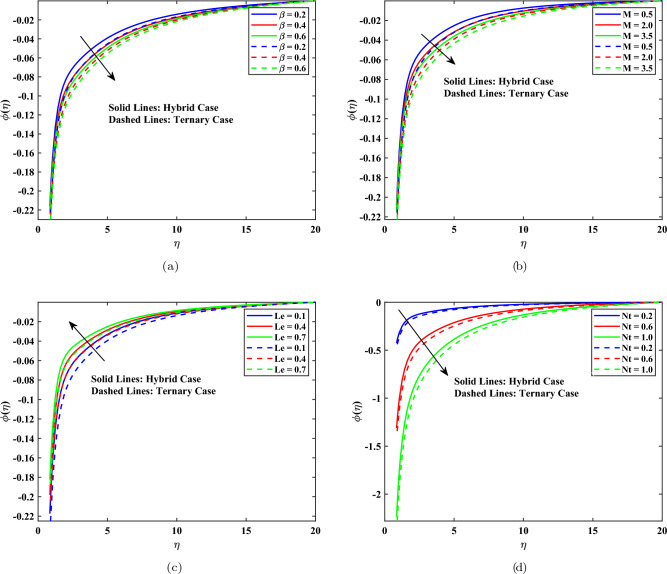
Concentration profile influenced by different parameters.

Figure 6 demonstrates the impact of physical parameters on the dimensionless concentration profile of hybrid and ternary nanofluids, that is, on the profile of $\phi (\eta )$. In Fig. 6(a), there is an increase in concentration distribution with an increase in Casson fluid parameter, which is represented by the value of $\beta$. This can be attributed to the heightened stress in the form of a yield, causing inhibition of convective mass flow, leading to a larger aggregation of nanoparticles at the surface. As shown in Fig. 6(b), a greater concentration profile with a larger magnitude of magnetic parameters *M* is due to the Lorentz force. This slows the velocity of the fluids and enhances the concentration boundary layer. Figure 6(c) shows that the concentration profile and thickness of the boundary layer decrease with the increase in the Lewis number, *Le* because increasing the Lewis number leads to a decrease in mass diffusivity. As shown in Fig. 6(d), the thermophoresis parameter Nt significantly increases the concentration field by transporting nanoparticles between low and high temperature regions, causing the increase in species concentration. In every case, the ternary nanofluid had slightly higher concentrations than the hybrid nanofluid, which indicated the enhancement of mass transfer due to the contact of multiple nanoparticles. The uniform decay of concentration profiles confirms the validity of the developed conditions of boundaries.

### Entropy generation and Bejan numbers

**Fig. 7:**
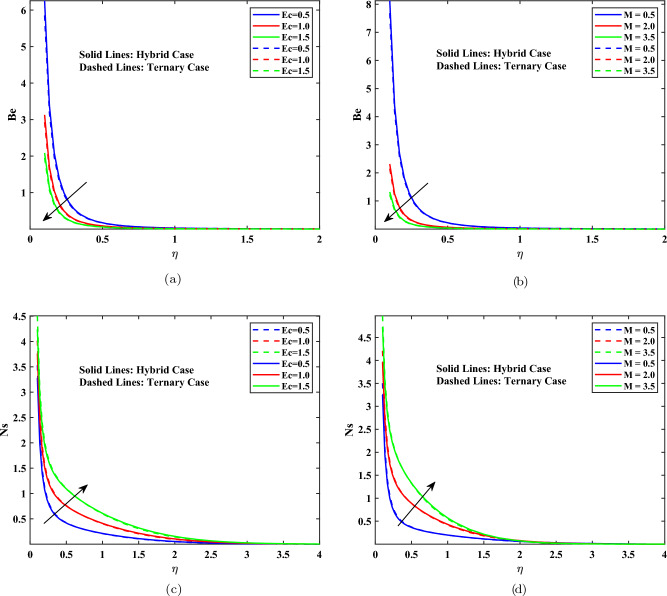
Profiles influenced by Eckert number, magnetic parameter, Bejan number, and entropy generation.

**Fig. 8:**
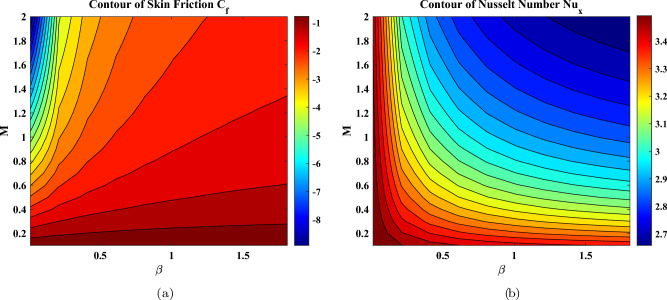
Contour plots for (**a**) skin friction and (**b**) Nusselt number.

Entropy formation quantifies thermal irreversibility inside a system. whereas the Bejan number *Be* measures the ratio of the amount of entropy produced by thermal transport to the total amount of entropy produced. The Bejan number is used to measure the interaction between the transmission of heat and the irreversibilities of viscous nature. A shift in $Be\approx 0$ to $Be\approx 1$ indicates a shift in thermally driven to friction-dominated entropy production. This change is determined by the interaction of the temperature gradient and velocity gradient within the boundary layer. Such parameters as the magnetic parameter, Casson parameter, and Brinkman number are critical parameters that have significant influence on this equilibrium and can be of interest as far as thermodynamic optimization is concerned. According to the 2nd law of thermodynamics, the total entropy of a closed system is always positively increasing in any real procedure; thus, the production of entropy is a positive quantity by definition. Figure 7 shows how the Eckert number *Ec* and magnetic parameter *M* affect the Bejan number *Be* and entropy generation rate *Ns* for hybrid and ternary nanofluids, respectively. Figures 7(a) and (b) demonstrate raising *Ec* and *M* reducing the Bejan number. As viscous dissipation (higher with larger Ec) and magnetic resistive forces (higher with larger M) increase, fluid friction becomes the dominant mode of irreversibility in the flow system, surpassing heat transfer. As a result, the *Be*, represents the ratio of irreversible heat transfer to total irreversibility falls. Figures 7(c) and (d) demonstrate that raising (*Ec*) and *M* leads to a large rise in total entropy production (*Ns*). This is owing to enhanced viscous dissipation generated by a greater Eckert number, along with Joule heating and Lorentz force-induced dissipation from a stronger magnetic field. A combination of these aspects contributes to the thermodynamic irreversibility of the system that promotes the overall production of entropy. The comparative analysis between hybrid and ternary nanofluids in the figures illustrates the effect that the nanoparticle structure has on the reactivity of Be and Ns to these parameters, where ternary nanofluids tend to respond more prominently because they have a modified thermal and electrical behavior. Figure 8 indicates that in an increase in both magnetic parameters *M* and Casson fluid parameter $\beta,$ the skin friction coefficient $C_f$ changes approximately between -8 and -1, which means that there is a very strong increase in the magnitude of the wall shear at higher values of the two parameters. Conversely, the Nusselt number $Nu_x$ is reduced, a non-negligible difference in heat transfer. The greatest heat transfer is at low *M* and low values of $\beta$ whereas the maximum shear stress is experienced at high values of both.

## Artificial neural network modeling

This work came up with a trained feed-forward ANN model to forecast the important thermal transport parameters. The model was aimed at replicating the integrated system of biological neurons. The data used to make forecasts was obtained based on a set of equations that were numerically solved to obtain the governing physical equations. The Neural Network Toolbox in MATLAB was used to develop the model and train it. The Levenberg-Marquardt algorithm of backpropagation was used in the training process because it is known to be efficient in the solution of nonlinear regression. The input layer comprised eight neurons, aligned with the primary non-dimensional parameters of the model: magnetic parameter *M*, radiation parameter *Rd*, nanoparticle shape factor *m*, Eraket number *Ec*, thermophoresis parameters *Nt*, Casson fluid parameter $\beta$, needle parameters *c*, and Lewis number *Le*. The output layer included two neurons denoting $Nu_x Re_x^{-1/2}$ and $C_f Re_x^{1/2}$. A sensitivity analysis involving networks showed that the architecture of the model was only needed to have one hidden layer. The tansig (hyperbolic tangent sigmoid) activation function was used in this hidden layer to identify the optimum arrangement of 10 neurons. A transfer function that was applied in the output layer was purelin (linear). This artificial neural network (ANN) model was designed to estimate the thermal and flow properties of a hybrid nanofluid, and the data obtained by the Bvp4c numerical solver was used as the training and validation input. Sufficient coverage of the input parameters was provided by generating 1000 different data through a Latin Hyperbole Sampling technique. The dataset was split into three subsets (training 70%, validation 15% and testing 15%) to simplify the process of model creation. Pre-training standards of input and output variables were made as it allowed the convergence of the neural network to be made constant and quick. Although the present research is comparatively commendable in terms of the need to demonstrate how a feed-forward artificial neural network can be employed and successfully utilized in this particular forecasting task, it is essential to note the bigger picture of data-driven methods. Further research could be conducted on the performance of alternative or hybrid machine learning approaches. The Support Vector Machines can be used due to their accuracy in the high-dimensional spaces, Gaussian Process Regression to predict probabilities and quantify uncertainties, and Physics-Informed Neural Networks, which incorporate the laws of physics in the learning process to make them more interpretable and efficient in utilizing data. These mathematical models enhance the physical interpretation, prediction generalization and uncertainties in complex nanofluidic systems. The information is collected and the output layer keeps it and has connections to allow its flow through the input layer to the next levels and corrections take place based on the error rate between the desired and projected value. The use of backpropagation to relay data to the input layer is aimed at minimizing errors. The training of the artificial neural networks is highly dependent on efficient data processing. A limited data set is a barrier to the network to find the correlation between the variables and, as a result, inadequate generalization. On the other hand, a very large or excessive data can create chances of overfitting and high processing requirements. Thus, it is critical to select an adequate and representative dataset in order to achieve the best results in artificial neural networks.

**Fig. 9:**
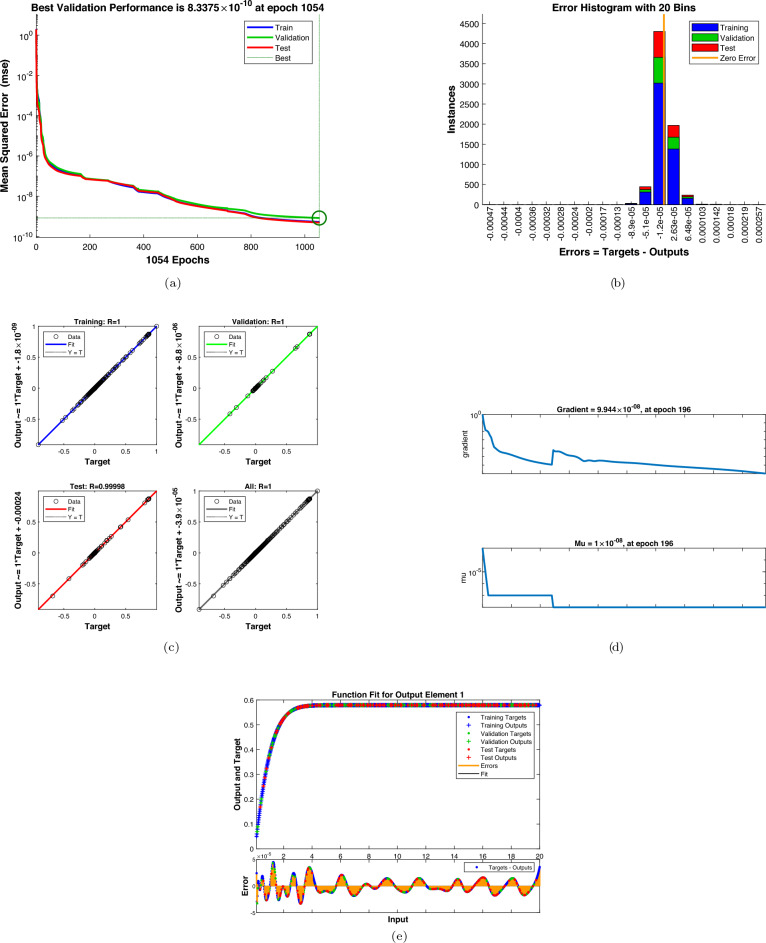
The ANN training results for the magnetic parameter (**a**) mean squared error; (**b**) error histogram; (**c**) regression analysis (**d**) training states; and (**e**) function fit.

**Fig. 10:**
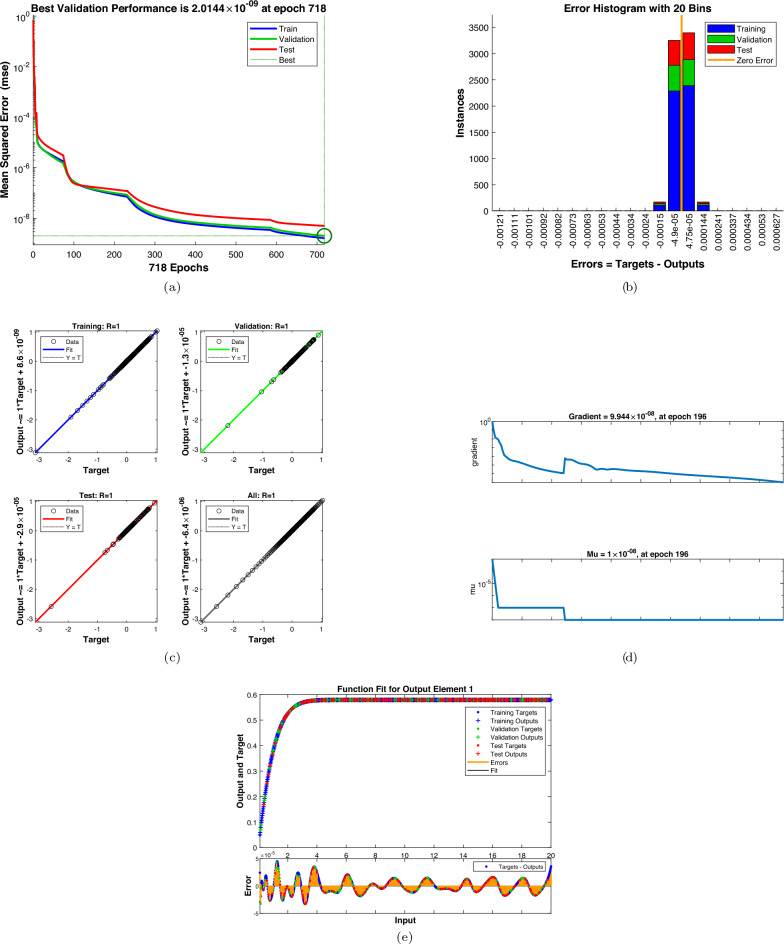
The ANN training results for the radiation parameter (**a**) mean squared error; (**b**) error histogram; (**c**) regression analysis, (**d**) training states; and (**e**) function fit.

As shown in Figs. 9 and 10, the Artificial neural network training process with respect to dimensionless parameters involves training performance indicators which include regression plots, autocorrelation analysis, functional fit, training state evolution, and the mean squared error reduction. The training techniques were able to decrease the MSE per epoch to a plateau which indicated that the best possible learning capacity had been reached, and no further significant improvement could be achieved by an extra round of training. The accuracy and predictability of the built artificial neural network model is demonstrated in Fig. 9 that indicates the influence of the magnetic parameter, M, on the velocity profile of the fluid mass that flows around a small needle. The latter reduces very fast in the initial epochs, and then it approaches a minimum of $10^{-6}$. The highest validation performance is found at epoch 1054. The similarity between the training and validation curves and testing curve shows that there is uniform learning and no overfitting. The histogram of errors in Fig. 9(b) shows the distribution of error between ANN prediction and target numerical data. The largest number of errors are densely clustered about zero and a huge proportion of examples lie within a small range. This distribution of zero showing symmetry can be used to demonstrate the absence of systematic bias and point to high accuracy of ANN predictions of training, validation, and testing datasets. Regression graphs of training, validation, testing, and total datasets are depicted in Fig. 9(c). The outputs of ANN are nearly perfectly linearly correlated to numerical targets as say by a correlation coefficient of approximately unity in each instance. The size of this large link indicates that the ANN was able to learn the underlying functional mapping that governs the behavior of fluid flows. Figure 9(d) demonstrates how gradient and adaptive learning parameter changes through the course of training. The actual decrease of the gradient is an indication of minimization of the cost, and the actual decrease of the parameter of decrement, continuous decrease of the Levenberg-Marquardt optimization process. It was found that convergence could be obtained in a few epochs, which depicts the efficiency of the ANN framework in terms of computation. In ref Fig. 9(e), the function fit plot shows that ANN predictions and actual outputs have a strong correlation across the input domain. The associated error signal is limited to small oscillations, which proves the resiliency of the trained network and its predictive power. The ANN model has good accuracy, better convergence rate and great generalization abilities making it a good surrogate model in predicting the thermofluid properties of the hybrid nanofluid flow system. The ANN training output of the radiation parameter in the figure below, Fig. 10, and the temperature profile show amazing convergence which is defined by a rapid reduction in mean squared error with the increase in the number of epochs. As shown in Fig. 10(a), the mean squared error significantly reduces within the first training process and slowly approaches the lowest value of 2.01 times $10^{-9}$ and the highest validation performance is obtained at epoch 718. The correlation between the training, validation, and testing curves is good, which implies that the ANN model is neither overfitting the learning nor does it show irregular learning. The error histogram Fig. 10 in (b) shows that the temperature predictions are relatively accurate at different levels of radiations and most of the errors are close to zero. The close balance of the errors implies that there is no systematic bias in the ANN output and thus the performance of the ANN is consistent in all the datasets. Figure 10(c) in the appendix shows that regression analysis shows that the artificial neural network (ANN) temperature forecasts and the target values have a strong relationship, with linear concordance with correlation coefficients approaching one. The fact that data points follow the 45 morning line depicts the ability of ANN to perfectly reproduce temperature responses to changes in the radiation parameter. Figure 10(d) shows that the graphs of the training state have a steady decreasing gradient, which is supported by the adaptive control of the learning parameter. This makes the model converge numerically and optimally. The use of early stopping through validation tests would be a good way of reducing the risk of overfitting and still achieve an acceptable predictive accuracy. The role of the ANN output is associated with the real temperature values, and the figure above, namely, Fig. 10e, demonstrates the possibility of the network to generalize the complex thermal dynamics of radiative heat transfer. ANN model has been proved to be reliable and efficient when predicting temperature profiles of hybrid nanofluid flows that are influenced by radiation.

## Comparison btween ANN and Bvp4c results

**Fig. 11:**
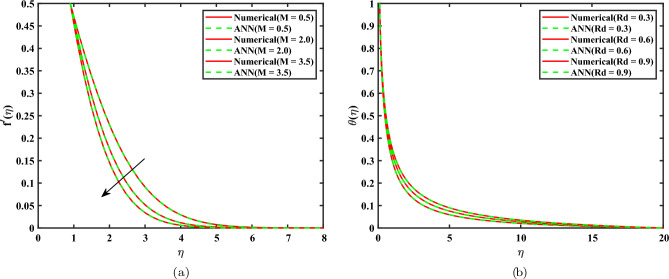
Comparison of Bvp4c and ANN for various parameters (**a**) Velocity for *M*; and (**b**) Temperature for *Rd*.

Figure 11(a) shows that $f'(\eta )$ changes with the similarity variable $\eta$ for verious values of the magnetic parameter $M$. This was done using both the numerical Bvp4c solver and the ANN model. The data show that higher $M$ causes the velocity reduction significantly across the entire boundary layer. The intensified magnetic field creates a stronger force, acts as a resistive drag force and slows down the fluid’s speed. Because of this, the thickness of the momentum boundary layer gets smaller as $M$ gets bigger. The ANN framework correctly reproduces the magnetohydrodynamic impact on the velocity field, as proven by the strong agreement between its predictions and the Bvp4c findings for all investigated $M$ values. Figure 11(b) indicates the impact of the radiation parameter which is denoted as *Rd* on $\theta (\eta )$. As *Rd* grows, the temperature within the boundary layer rises, and the thermal boundary layer gets thicker. This tendency is linked to the intensification of thermal radiation, which increases the effective thermal diffusivity, enabling the fluid to retain more heat and lowering heat loss from the surface. Additionally, the temperature profiles projected by the artificial neural network correlate precisely with the numerical results found with Bvp4c across the entire $\eta$ range, proving the ANN model’s capacity of accurately imitating the nonlinear properties of radiative heat transfer.

## Conclusion

A study employing numerical methods and artificial neural networks to analyze MHD Casson hybrid and ternary nanofluid flow over a heated slender needle has been successfully carried out.

- The magnetic parameter M diminishes the fluid velocity by strengthening the Lorentz force, leading to a reduction in the thickness of the momentum boundary layer.
- An increase in the radiation parameter $R_d$ greatly improves the temperature profile $\theta (\eta )$ by increasing the effective thermal diffusivity, resulting in a larger thermal boundary layer.
- Casson fluid, needle, and magnetic characteristics drastically restrict velocity owing to increased viscosity and Lorentz force effects.
- Thermal radiation, viscous dissipation, thermophoresis, and nanoparticle form factor all significantly improve the temperature field and thermal boundary layer.
- Ternary nanofluids routinely outperform hybrid nanofluids in terms of momentum and heat transfer due to synergistic nanoparticle interactions.
- Entropy production rises with the Eckert number and magnetic strength, but the Bejan number implies a shift toward friction-dominated irreversibility.
- ANN predictions correspond well with numerical data, demonstrating remarkable accuracy and strong generalization potential.
- The consistency between ANN predictions and Bvp4c solutions confirms the ANN as a credible surrogate model for complicated magneto-radiative thermo-fluid dynamics.

## Data Availability

The data that support the findings of this study are available from the corresponding author upon reasonable request.

## List of symbols

$$\eta$$: Similarity variable
$f(\eta )$: Dimensionless stream function
$$\phi (\eta )$$: Dimensionless concentration
*Nu*: Nusselt number
$\beta$: Casson fluid parameter
*c*: Needle (size) parameter
*Pr*: Prandtl number
*Ec*: Eckert number
*Nt*: Thermophoresis parameter
*Be*: Bejan number
$$k_{thnf}$$: Thermal conductivity
$\rho _{thnf}$: Density of try-hybrid nanofluid
$$u,\, v$$: Velocity components ($$\text {m s}^{-1}$$)
$$\theta (\eta )$$: Dimensionless temperature
$C_f$: Skin friction coefficient
*Sh*: Sherwood number
*M*: Magnetic parameter
*m*: Nanoparticle shape factor
*Le*: Lewis number
*Rd*: Thermal radiation
*Nb*: Brownian motion
*Ns*: Entropy generation number
$$\mu _{thnf}$$: Dynamic viscosity
$(\rho c_p)_{thnf}$: Heat capacity of try-hybrid nanofluid
